# From child and parent needs to supportive interventions for proton therapy without anaesthesia: A qualitative study^☆^

**DOI:** 10.1016/j.tipsro.2026.100441

**Published:** 2026-09-27

**Authors:** Pernille Bekke, Yasmin Lassen-Ramshad, Britta Weber, Klaus Seiersen, Anne Wilhøft Kristensen

**Affiliations:** Danish Centre for Particle Therapy, Aarhus University Hospital, Aarhus, Denmark

**Keywords:** Proton therapy, Anaesthesia, Paediatrics, Anxiety, Coping, Qualitative research

## Abstract

**Introduction:**

Children must remain still during proton therapy (PT) to ensure precise treatment delivery. For some children, distress, separation from parents, or difficulties coping with the treatment situation necessitate treatment under anaesthesia. This study aimed to explore children's and parents' experiences of PT delivered with or without anaesthesia, to inform the development of supportive interventions in paediatric PT intended to reduce the use of anaesthesia.

**Methods:**

Participant observations informed the development of interview guides. Children were interviewed using photo elicitation, and parents were interviewed using semi-structured interviews. Data were analysed using thematic analysis.

**Results:**

Five children and eight parents from six families participated. Anxiety emerged as a central experience, although with different expressions for children and parents. Children described anxiety as tied to the treatment situation, whereas parents described anxiety related to separation, uncertainty, and anaesthesia. Four themes were identified to help manage anxiety: control, predictability, information, and care through relationships and continuity. The findings suggested that awake treatment may be better supported when these needs are addressed. These findings informed the development of a targeted intervention consisting of preparation and information, exposure and training, and care and continuity.

**Conclusion:**

PT was experienced as demanding by children and parents, but anxiety did not appear to preclude awake treatment. Rather, the findings suggest that treatment-related anxiety became more manageable when needs for control, predictability, information, and care through relationships and continuity are addressed. These findings provide the basis for developing interventions in paediatric PT to reduce the use of anaesthesia.

## Highlights

• Anxiety was central but manifested differently in children and parents.
• Four needs supported awake treatment: control, predictability, information and care.
• Meeting these needs may make treatment-related anxiety more manageable.
• Findings informed three intervention domains to support awake proton therapy.

## Introduction

Anaesthesia is often required during radiotherapy for young children to ensure treatment precision and safety, and to reduce distress related to the treatment procedure and separation from parents [1], [2], [3]. Anaesthesia is generally safe but carries a risk of side effects such as airway obstruction, hypoxia and bradycardia. It also requires fasting and prolonged hospital stays. [3], [4], [5], [6], [7], [8]. Recent research has raised concerns that repeated exposure to anaesthesia may be associated with long-term neurocognitive outcomes [3], [9], [10].

Studies of children undergoing radiotherapy and cancer treatment show that treatment can be challenging and involve anxiety, discomfort, and side effects. However, preparation has shown to support children in coping and feeling safe during treatment. Preparation includes information, practice, parental presence, distraction, play, trust, individualised care, and supportive relationships with healthcare professionals (HCPs). Parents similarly express a need for adequate information and trusting relationships with HCPs [11], [12], [13], [14], [15], [16].

Previous studies have demonstrated that preparing children for radiotherapy can reduce the need for anaesthesia in children aged 3–4 years [3], [6], [17], [18], [19], [20], [21], [22]. However, there is limited knowledge of how children and their parents experience proton therapy (PT) delivered with or without anaesthesia, and what kinds of support may be important in this context.

This study aimed to explore children's and parents' experiences of paediatric PT delivered with or without anaesthesia. These experiences were used to identify support needs relevant to making awake treatment more feasible and to inform the development of interventions to reduce anaesthesia use in the Danish paediatric PT.

## Methods

This qualitative interview study explored children's and parents' experiences and preferences regarding PT delivered with or without anaesthesia at the Danish Centre for Particle Therapy (DCPT).

### Setting

DCPT is a national proton facility in Denmark. Administrative data obtained from the Danish National Research Centre for Radiotherapy indicate that approximately 35 Danish children require radiotherapy each year. Most are referred for PT, as this modality has been shown to reduce radiation exposure to surrounding healthy tissue and thereby potentially lower the risk of late effects, including secondary malignancies. This consideration is particularly relevant in paediatric patients with long life expectancy [23].

Children receive PT daily, five days a week, for several weeks. Daily treatment lasts 30 to 60 min. At DCPT, children up to the age of 8 are primarily treated under anaesthesia.

Parents and children receive information about PT from a paediatric oncologist and a radiation oncologist, and are given a booklet describing the radiotherapy process in a child-friendly format. At the clinic, they are met by a designated radiation therapy technologist (RTT). The RTT introduces the family to the treatment facilities and prepares a box containing a treatment calendar, stickers, beads for daily rewards, and, for some children, a teddy bear that can serve as a treatment buddy [24]. The RTT also explores the child's interests and facilitates individualised distractions.

### Participants and recruitment

Danish-speaking children (4–8 years) and parents were consecutively invited to participate during the child's treatment period at DCPT. All six invited families accepted participation.

### Data collection

Participant observations were conducted at DCPT to gain insight into the organisational context and clinical practice related to paediatric PT. The first author (PB), a nurse and RTT with in-depth knowledge of the clinical context, carried out these observations to inform data collection and analysis. Observations were conducted in the clinic the year preceding the interviews and focused on aspects such as flow, duration, immobilisation procedures, distraction strategies, and relational interactions between staff, children, and parents [25], [26].

Insights from the observations informed the development of probe questions for the child interviews and the interview guide for the parental interviews [27], [28]. For the child interviews, photographs of treatment procedures were used alongside probe questions to support recall and facilitate the expression of experiences and emotions [28], [29]. The child probe questions are presented in Supplementary Material 1, and the parental interview guide in Supplementary Material 2.

Interviews were conducted 1–5 months after completion of PT between February and September 2025. One child was interviewed five months after treatment due to limited capacity within the family. All interviews were audio-recorded, conducted individually, and performed by PB. Parents and children were present in the room during interviews. In one child interview, a parent intervened, and the affected part of the interview was excluded from the analysis. Child interviews lasted 25–45 min and parent interviews 30–75 min.

### Data analysis

Interviews were audio-recorded, transcribed, and imported into NVivo 15 for data management [30], [31]. Data were analysed using reflexive thematic analysis following Braun and Clarke [32]. The analysis was primarily inductive in coding, while acknowledging that PB's clinical knowledge and prior observations informed the interview guides and interpretation of the data. After multiple readings of the transcripts, PB generated initial codes relevant to the research aim. Child and parent interviews were initially analysed separately to preserve the specificity of each perspective. Candidate themes were developed iteratively by examining patterns of shared meaning across codes, with codes contributing to more than one candidate theme where analytically relevant [32]. In a subsequent interpretive comparison, the two datasets were analysed together to explore similarities, differences, and overarching patterns across children's and parents' experiences. Throughout the analytical process, codes and candidate themes were discussed with the last author (AWK), a qualitative researcher and clinical nurse specialist at DCPT, and themes were iteratively reviewed and refined. Tables illustrating the analytic progression from initial codes to candidate and final themes for children and parents are provided in the supplementary material (Supplementary Tables 3 and 4).

### Ethical considerations

The project was submitted to the Central Denmark Region Committees on Health Research Ethics (reference no. 1–10–72-116-25) to determine whether ethical approval was required and to ensure compliance with applicable regulations. The study was deemed exempt from formal ethical approval.

Written informed consent was obtained from parents before the observations. For the interviews, parents were informed during the treatment course and provided consent. After treatment, families were contacted by telephone to confirm their willingness to participate, and written consent was obtained again on the day of the interview. Children provided oral assent during treatment and again before the interview.

Interviews were conducted in the families' homes to provide a familiar and secure environment for the children. This approach also meant that PB entered the child's home as a guest, intended to support a more balanced researcher-child relationship and reduce power asymmetry between the researcher and the child.

## Results

Five children and eight parents from six families participated. One child rejected participation on the day of the interview. The children were aged 4 to 8 years and diagnosed with CNS or solid tumours. One child underwent anaesthesia throughout treatment, three experienced both anaesthesia and awake treatment, and one completed treatment fully awake. Five mothers and three fathers were interviewed.

Across both datasets, treatment-related anxiety emerged as an overarching analytical finding, although with different expressions and meanings for children and parents. Importantly, anxiety did not in itself appear to prevent awake treatment; rather, awake treatment seemed more feasible when anxiety could be made manageable. Through an interpretive comparison of the two analyses, four interrelated themes were identified: control, predictability, information, and care through relationships and continuity. Together, these themes captured the support needs that appeared central to making anxiety manageable and thereby supporting awake treatment during paediatric PT.

### Anxiety as a shared but differently expressed experience

Children described anxiety as part of undergoing PT, especially in relation to entering the treatment room, wearing the treatment mask, unfamiliar sensory impressions during irradiation, and being alone during treatment. However, their descriptions suggested that this anxiety was often manageable rather than overwhelming. One child said: “I was a bit afraid, but still I think that it was ok” (ITB05). Their anxiety was described in concrete terms and was closely tied to what happened in the moment.

Parents also described anxiety as central, but their accounts suggested that it was broader and more existential in nature. It was particularly related to separation during treatment, uncertainty about treatment and outcomes, and concerns about anaesthesia and its effects on the child. One parent stated: “Every time my child had anaesthesia and closed its eyes, I felt like my child had died” (ITV04), while another said, “I would prefer to get radiation myself to be able to stay” (ITV03). Parents' accounts pointed to an emotionally demanding sense of responsibility that extended beyond the treatment moment itself.

Across both groups, awake treatment was generally preferred when the child was sufficiently prepared, supported, and able to cope with the treatment situation. Children who had experienced both awake treatment and anaesthesia preferred being awake when possible, largely because anaesthesia was associated with side effects. One child said, “When I had the sleeping medicine, I felt so much nausea when I woke up. I preferred to be treated while awake” (ITB05). Parents similarly preferred awake treatment when conditions allowed, although they also described anaesthesia as necessary in some situations to ensure safe treatment. Overall, these accounts suggest that the key issue was not the absence of anxiety, but whether anxiety could be made manageable. The following four interrelated themes capture the needs that appeared central to making anxiety manageable and thereby supporting awake treatment.

### Control: being able to act in the situation

Children described coping better with treatment when they were involved and had opportunities to influence what was happening. Hand signs, distraction, comfort objects, and small coping tools helped them maintain a sense of control. As one child explained, *“We had hand signs, and if I waved, they would come in” (ITB05)*. Another said, *“I could push onto the magic stone, and the smell would disappear” (ITB06).* These accounts suggested that control was rooted in the child's ability to participate, cooperate, and use concrete strategies to manage the treatment situation.

For parents, the need for control was expressed as a need to feel reassured that the child could manage the situation and that the child could be handed over safely to HCPs. This was especially visible when parents described relief after seeing the child cope successfully while awake. One parent said, *“My child tried to lie on the treatment bed… after that, I had no doubt that my child could be treated awake” (ITV07)*. Parents' sense of control thus appeared to depend on whether the child seemed able to participate and cooperate, and whether the treatment situation felt manageable and safe.

### Predictability: knowing what will happen

For children, familiar routines and repeated procedures were described as calming because they made the treatment situation recognisable. One child explained, *“It was always the same… it was nice that they did things the same way, so I knew how it would be” (ITB03*). Children's accounts also suggested a need for predictability in the overall treatment trajectory, as understanding where they were in the process and how many treatment fractions remained made the course feel more manageable.

Parents associated predictability with reduced anxiety. Their accounts pointed to the importance of routines, preparedness, and a well-organised treatment and care pathway. One parent described reassurance in knowing that *“Even before we entered the centre, there was a plan. We were welcomed, and staff knew exactly what to do” (ITV02).* Over time, familiarity with routines and observing the child's increasing confidence also seemed to reduce parental anxiety: *“As days passed, it got easier. My child took ownership and knew what was coming” (ITV05).*

### Information: understanding the situation and regulating uncertainty

For children, information seemed important when it was concrete and closely linked to what they would experience in the treatment room and during related procedures. Their accounts suggested that such information reduced uncertainty and helped make treatment-related anxiety manageable.

For parents, information played a more explicit role in regulating trust in HCPs and reducing uncertainty. Parents described accurate, timely, and consistent communication as essential to feeling safe, while inconsistent information increased distress. One parent stated*, “You are in a crisis, and the last thing you need is inconsistent information” (ITV04).* At the same time, parents differed in how much information they wanted. Some wanted detailed explanations, while others found too much information overwhelming: *“It was overwhelming. I would have preferred to have had less information. You can't comprehend it at that time” (ITV02).* Daily feedback from HCPs was experienced as particularly reassuring. As one parent put it*, “It felt safe that they came every day after treatment and told me how it went” (ITV01).* These findings suggest that information did not merely transfer knowledge but also helped regulate parental uncertainty and trust.

### Care through relationships and continuity: feeling known, seen, and supported

Children's and parents' accounts also pointed to a need for care that was supported by relationships with HCPs, familiarity, and continuity. For children, this was reflected in the importance of feeling safe with staff, being listened to, and being supported by individualised distractions and comforting routines. Their accounts suggested that anxiety became manageable when the treatment setting felt relationally safe.

Parents described this need more explicitly as being recognised as persons and as staff knowing their child and understanding what helped. One parent noted, *“I don't know if it is written in my child's medical journal... but it was very obvious that everybody knew what helped” (ITV06).* Relational continuity appeared to reassure parents not simply because the same staff were present, but because care was experienced as coherent, attentive, and tailored to their child.

### From identified needs to intervention design

The four identified support needs provided the empirical basis for developing initial intervention components intended to make awake treatment more manageable. The findings were discussed within an interprofessional paediatric group at DCPT, which translated the identified needs into clinical strategies and materials drawing on clinical experience. The resulting components included newly developed materials as well as existing supportive practices that were further developed and structured (Table 1). These initial components are currently being iteratively tested and refined in clinical practice.

**Table 1:** Intervention components, core content, development status, and support needs addressed.

Intervention component	Core content	Development status	Support needs addressed
Preparation and information	• Procedural picture book • Department tour of treatment, scan and mould rooms • Parent guidance in supporting the child during radiotherapy	Existing practice, further developed	• Predictability • Information • Relationship/care
	• Animated film about radiotherapy • Teddy bear scan with an inserted Lego brick to show the child how images are used • Child-centred “who are you” questionnaire	Newly developed	
Exposure and training	• Participation of hospital clowns during first visits, practice sessions and early treatment sessions to support relational continuity, play-based exposure and coping	Existing practice, further developed	• Control • Predictability • Relationship/care
	• Introduction to home practice in lying still and coping with separation before the first visit • One to three treatment practice sessions in the department with RTTs • Use of a play gantry	Newly developed	
Care and continuity	• Designated treatment team • Treatment calendar with stickers to show progress	Existing practice	• Relationship/care • Predictability • Control
	• Standardised routines and procedures • Individualised care and coping strategies, including distraction during treatment with movies, music or audiobooks	Existing practice, further developed	
	• Child's own suitcase with gadgets, and daily rewards	Newly developed	

## Discussion

In this study, anxiety emerged as a central experience in paediatric PT for both children and parents. However, the findings also suggest that anxiety did not necessarily preclude treatment without anaesthesia. Rather, treatment without anaesthesia appeared to depend on whether anxiety could be made manageable through support addressing children's and parents' needs for control, predictability, information, and care through relationships and continuity. The study therefore identifies needs that may support treatment without anaesthesia.

Previous studies have identified anxiety as a significant challenge in paediatric radiotherapy and highlighted the importance of preparation, familiarity with the treatment setting, distraction, play, and supportive relationships with HCPs [3], [6], [12], [14], [16], [17], [21]. Our findings support this literature, but add that preparation, practice, distraction, and relational support may help children understand what will happen, become familiar with the treatment situation, and participate more actively during treatment. In our study, children's anxiety was concrete and situational, related to the treatment room, treatment mask, sensory impressions, and being alone during treatment. Thus, support that helps children understand the treatment situation, practice lying still, use distraction, and signal when they need help may contribute to making anxiety manageable.

Parents described a broader form of anxiety related to separation, uncertainty, anaesthesia, and responsibility for their child's wellbeing. This aligns with previous research showing that parental anxiety may involve concerns about the child's suffering, treatment experiences, and wider illness trajectory [12], [14]. Our findings add that parental reassurance was closely linked to trust in the care environment and confidence that the child was known, supported, and able to cope. Preparation alone may therefore be insufficient without relational continuity, coherent routines, responsive communication, and a consistent point of contact [13].

Taken together, these findings point to the interdependence of children's and parents' experiences. Children's coping appeared closely linked to parents' need for reassurance, trust, and confidence in the surrounding care, while children's visible coping seemed to reduce parental anxiety. Support during PT should therefore be understood not only as preparing the child, but as a relational, family-oriented care process [33], [34]. This resonates with child-centred care perspectives, where involvement means creating opportunities for meaningful participation [35]. In our study, this was reflected in hand signs, routines, distractions, comfort objects, and coping tools that allowed children to act within the situation.

This study has limitations. The sample was small and drawn from a single centre, which may restrict transferability. Recall bias may also have influenced participants' accounts, as interviews were conducted 1–5 months after completion of PT, and social desirability bias may have affected responses, as interviews concerned care received at the treating centre. In addition, children and parents were present in the same room during interviews, which may have introduced a spillover effect between their accounts, although this setting may also have supported children's sense of safety. Including both perspectives strengthened the analysis by illuminating how anxiety and support needs were interconnected across the family context.

The findings have important clinical implications. They do not demonstrate the effectiveness of a specific intervention but identify central needs and a potential mechanism that informed the development of the intervention. More specifically, the findings suggest that anxiety may become more manageable when care addresses children's and parents' needs for control, predictability, information, and care through relationships and continuity. On this basis, a targeted intervention was developed for paediatric PT, consisting of preparation and information, exposure and training, and care and continuity.

The intervention is being feasibility-tested in clinical practice using an iterative, participatory design, in which HCPs, parents, children, and researchers contribute to its ongoing adaptation and refinement.

## Conclusion

PT was experienced as demanding by both children and parents, but anxiety did not appear to preclude awake treatment. Rather, the findings suggest that treatment-related anxiety became more manageable when support addressed needs for control, predictability, information, and care through relationships and continuity. These findings provide an empirically grounded basis for developing supportive interventions for children and parents during paediatric PT. On this basis, a targeted intervention was developed, consisting of preparation and information, exposure and training, and care and continuity, and is now being feasibility-tested in clinical practice.

## Declaration of generative AI and AI-assisted technologies in the manuscript preparation process

During the preparation of this work, the authors used Grammarly and ChatGPT (Open AI) to support language refinement, readability, and structuring of manuscript-related text. After using these tools, the authors critically reviewed, revised, and approved all content and take full responsibility for the final manuscript.

## CRediT authorship contribution statement

**Pernille Bekke:** Writing – review & editing, Writing – original draft, Validation, Project administration, Methodology, Investigation, Formal analysis, Data curation, Conceptualization. **Yasmin Lassen-Ramshad:** Writing – review & editing, Conceptualization. **Britta Weber:** Writing – review & editing, Conceptualization. **Klaus Seiersen:** Writing – review & editing, Conceptualization. **Anne Wilhøft Kristensen:** Writing – review & editing, Writing – original draft, Visualization, Supervision, Project administration, Methodology, Formal analysis, Conceptualization.

## Funding

This research received no specific grant from funding agencies in the public, commercial, or not-for-profit sectors. The study was conducted as part of clinical development and research activities at the Danish Centre for Particle Therapy, Aarhus University Hospital.

## Declaration of competing interest

The authors declare that they have no known competing financial interests or personal relationships that could have appeared to influence the work reported in this paper.

## Data Availability

The qualitative data supporting the findings of this study are not publicly available due to the sensitive nature of the data and the involvement of children and families. Data may be made available upon reasonable request to the corresponding author, subject to relevant ethical and data protection considerations.

## Appendix A Supplementary data

Supplementary material 1 Children’s probe questions.
