# Supplementary material for: From child and parent needs to supportive interventions for proton therapy without anaesthesia: A qualitative study
Source: Tech Innov Patient Support Radiat Oncol. 2026 Sep 27;40:100441. doi: 10.1016/j.tipsro.2026.100441 (PMC13654558; doi:10.1016/j.tipsro.2026.100441)
Supplement: Supplementary file 1 [file mmc1.docx]

Supplementary 1. Children’s probe questions

| Preparation | Can you tell me how it was to come to DPCT? |  |
| --- | --- | --- |
|  | How was it to have the mask made? | How was it to have the mask on during treatment? |
|  | Did we tell you what radiotherapy was alle about? | Do your think there was anything we said that did not fit? |
|  | Did we listen to you? |  |
|  | Was there anything that made it ok to come to us? | Clowns, distraction, rewards? |
|  |  |  |
| Anaesthesia | How did it feel to get sleeping medication? |  |
|  | Is there anything you think we should do to make it easier for other children who need sleeping medication |  |
| Awake | Tell me how it felt to be awake during treatment? |  |
|  | Is there anything you think we could do to make it easier to stay awake during treatment? | Distractions? |
|  | Did we ask you how you were? | Hand signs |
|  | Were you scared while lying in the room? |  |
|  | Did you see any light or smell something? | What was it like? |
| Anaesthesia or awake | What did you find best sleeping or being awake? |  |
|  | If one of your friends are going to have radiotherapy- do you think that one should sleep or be awake? |  |

Photo 1	Photo 2	Photo 3	Photo 4

*Photos: Maria Thorndahl & Pia Randers*
