# Supplementary material for: From child and parent needs to supportive interventions for proton therapy without anaesthesia: A qualitative study
Source: Tech Innov Patient Support Radiat Oncol. 2026 Sep 27;40:100441. doi: 10.1016/j.tipsro.2026.100441 (PMC13654558; doi:10.1016/j.tipsro.2026.100441)
Supplement: Supplementary file 2 [file mmc2.docx]

Supplementary 2. Parental interview guide

|  |  | **Probe question** |
| --- | --- | --- |
| Preparation | Can you describe your first visit in DPCT? | Worries?  Looking forward to? |
|  | How was your experience concerning being meet in needs and wishes? |  |
|  | Were you involved in deciding whether your child should be treated while awake or under anaesthesia? | Advantages?  Disadvantages? |
|  |  |  |
| Treatment day 1 | Can you describe the first day of treatment | Concerns?  Looking forward to?  Context? |
|  | How did you experience leaving your child alone in the room during treatment? |  |
|  | How did you experience that your needs and wishes were fulfilled? |  |
|  |  |  |
| If child was treated under anaesthesia | How did you experience your child to have to go under anaesthesia |  |
|  | How have you experienced the awakening of your child? | Reactions? |
|  | How was the rest of the day for your child after anaesthesia? | Reactions?  Side effects? |
|  |  |  |
| If your child was treated while awake | How did you experience your child being awake during treatment? | Advantages?  Disadvantages? |
|  |  |  |
| If the child was treated partly under anaesthesia and partly awake | How did you experience the difference between your child being treated under anaesthesia and awake? | Advantages?  Disadvantages? |
|  | Did you experience any changes during the course of treatment | Anxiety?  Relationship? |
|  | How would you have preferred your child to be treated | Anaesthesia?  Awake? |
| In general, | How did you experience the process in PT? | Have we been able to adapt treatment to your child/family?  Suggestions for improvement? |
