# Supplementary material for: From child and parent needs to supportive interventions for proton therapy without anaesthesia: A qualitative study
Source: Tech Innov Patient Support Radiat Oncol. 2026 Sep 27;40:100441. doi: 10.1016/j.tipsro.2026.100441 (PMC13654558; doi:10.1016/j.tipsro.2026.100441)
Supplement: Supplementary file 3 [file mmc3.docx]

Supplementary 3. Illustration of analytic procces for children

| **Initial codes** | **Candidate themes** | **Final themes** |
| --- | --- | --- |
| Teddy bear, music, stone, distraction | Distraction and self-regulation | Control |
| Hand signs, the ability to stop | Having a say |  |
| Nausea from anaesthesia | Bodily consequences |  |
| Smell, mask, being alone, anxiety, anaesthesia, awake | Treatment-related uncertainty | Predictability |
| Feeling safe, routine, communication | Predictability and safety |  |
| Calendar, stickers, rewards | Structuring time |  |
