# Supplementary material for: From child and parent needs to supportive interventions for proton therapy without anaesthesia: A qualitative study
Source: Tech Innov Patient Support Radiat Oncol. 2026 Sep 27;40:100441. doi: 10.1016/j.tipsro.2026.100441 (PMC13654558; doi:10.1016/j.tipsro.2026.100441)
Supplement: Supplementary file 4 [file mmc4.docx]

Supplementary 4. Illustration of analytic process for parents

| **Initial codes** | **Candidate themes** | **Final themes** |
| --- | --- | --- |
| Maturity, age, treatment duration, sensory impressions, able to lie still, anxiety, safety | The choice of treatment with or without anaesthesia | Control |
| Side effects, behavioural changes, change in daily routines | Consequences of being treated under anaesthesia |  |
| Energy, quality of life, mood, appetite | Experiences from the child being awake |  |
| Distractions, routines, play, predictability, training, relationships, control, and coping | Preparation for treatment |  |
| Anxiety, chaos, earlier experiences, uncertainty, side-effects | Treatment-related uncertainty | Predictability |
| Side effects: nausea, weight loss, fatigue, and uncertainty | Worries about the present and the future |  |
| Preparation, routines, trust, feeling safe | Anxiety level can be affected |  |
| Crisis, chaos, inconsistency, capacity, overwhelmed | Information creates a basis for trust or distrust | Information |
| Individual approach, feedback, trust, confidence | Feeling informed |  |
| Abandoned my child, letting go, separation | Child alone in the treatment room | Care through relationships and continuity |
| Calm, time, architectural setting, staff prepared | The context surrounding the treatment is soothing |  |
| Individual approach, relationship, preparation, trust, confidence, quality of life | They see our family |  |
| Quality of life, treatment times, choices, distraction, relationship, and having a say | Focus on my child |  |
